# Risk factors for refracture of the femoral shaft in children after removal of external fixation

**DOI:** 10.1186/s10195-021-00569-9

**Published:** 2021-02-24

**Authors:** Meizhen Guo, Yuxi Su

**Affiliations:** Department II of Orthopedics, Chongqing Key Laboratory of Pediatrics, Ministry of Education Key Laboratory of Child Development and Disorders, National Clinical Research Center for Child Health and Disorders, China International Science and Technology Cooperation Base of Child Development and Critical Disorders, The Children’s Hospital of Chongqing Medical University, 136# Zhongshan 2road Yuzhong District, Chongqing, 400014 China

**Keywords:** Femoral refracture, External fixation, Children

## Abstract

**Background:**

External fixation is the primary treatment option in children for femoral shaft fractures, such as open femoral or multiple fractures. One complication is refracture, which is the biggest limitation of fixation devices. This study aims to investigate the risk factors associated with refracture after the removal of external fixation devices and decrease the frequency of refracture.

**Materials and methods:**

Retrospectively reviewed clinical data of 165 patients treated at our hospital for fresh femoral shaft fractures with external fixation between May 2009 and February 2018 were included in this study. Patients with pathological fractures, fractures of the femoral neck, fractures that were fixed using plates or elastic stable intramedullary nailing, and old fractures, as well as those who underwent postoperative femoral surgery were excluded. Potential risk factors included: patient age, gender, and weight, fracture sides, open or closed fracture, fracture sites, reduction methods, operation time, perioperative bleeding, number and diameter of the screws, and immobilization time. These factors were identified by univariate and logistic regression analyses.

**Results:**

Femoral shaft refracture developed in 24 patients. Univariate analysis revealed that refracture was not statistically significantly associated with any of the above factors, except AO Pediatric Comprehensive Classification of Long Bone Fractures (PCCF) classification type 32-D/4.2 and L2/L3 ratio (L2, length of femur fixed by the two screws farthest from the fracture line; L3, the total length from the greater trochanter to the distal end of femur; *P* < 0.001 and *P* = 0.0141, respectively). Multivariate analysis showed that PCCF classification type 32-D/4.2 and L2/L3 ratio were also independent risk factors for femoral refracture.

**Conclusions:**

Femoral shaft refracture is relatively common in children treated with external fixation. Because of the limited number of cases in this study, we cautiously concluded that the PCCF classification type 32-D/4.2 and L2/L3 ratio were independent risk factors for femoral shaft refracture in these patients.

**Level of evidence:**

IV

## Introduction

Femoral shaft fracture is not rare, accounting for about 1.6–2% of all trauma accidents in children [1, 2]. The treatment of simple pediatric femoral shaft fractures is based on the patient’s age [3]. The American Academy of Orthopedic Surgeons and the Pediatric Orthopedic Society of North America [4, 5] suggest that treatment for femoral shaft fractures in children under the age of 5 years should involve a Pavlik harness, a spica cast, or skeletal traction. For school-aged children or even younger patients, elastic intramedullary nail (EIN) fixation is the first choice and becoming increasingly accepted by most surgeons [6–8]. However, external fixation (EF) may still be used when EIN is not a suitable option, such as with open fixation, multiple fractures, femoral fractures with severe skins lesions, patient weight over 50 kg, proximal or distal humeral fractures that EIN fixation cannot fix [9, 10]. In the above cases, EF was the first choice for fixation. In this study, we focused on patients who underwent EF in our hospital during the last 10 years.

Some complications may occur with EF that do not occur in EIN fixation, such as pin infection, impaired knee function, limitation of activity, poor cosmesis, or even refracture [9, 10]. Among the complications, refracture, which occurs with an incidence of 6.5–14.2%, is the most severe and may lead to further surgeries, prolonged rehabilitation times, or even litigation [1, 11, 12]. Some studies have reported that factors contributing to femoral shaft refracture include early fixator removal, open fracture, and open reduction [11, 13]. Fracture type has also been linked to refracture [13–15], but its significance remains unclear. Patient characteristics, such as sex, age, and weight, may also be associated with refracture risk. The related high-risk factors may help decrease the incidence of refracture and help the doctor stay out of trouble.

Here, we studied the risk factors of femoral shaft refracture after the removal of EF in children treated in our hospital to determine the high-risk factors for refracture in EF.

## Materials and methods

We retrospectively reviewed the clinical data of 165 children with femoral shaft fractures who were treated via EF in our hospital from May 2009 to February 2018. The inclusion criterion was femoral shaft fracture with follow-up time of more than 12 months. Patients with pathological fractures, fractures of the femoral neck, fractures fixed using plates or elastic stable intramedullary nailing, and old fractures, as well as those who underwent postoperative femoral surgery, were excluded. In this study, refracture means the fracture happened again in the same femur not more than one year since the first fracture. As this is a retrospective study, the STROBE checklist was adopted.

**Fig. 1:**
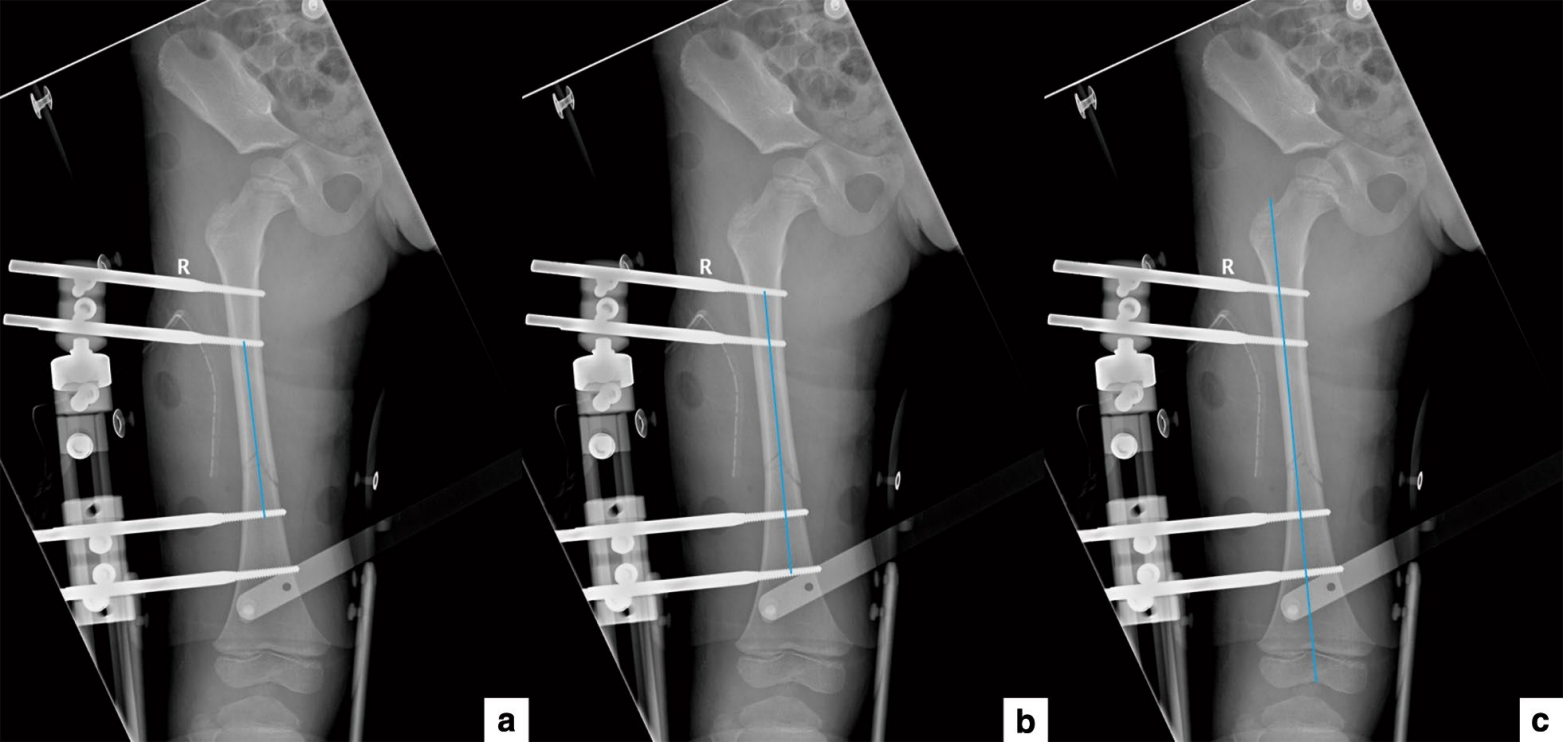
A diagram of L1, L2, and L3. **a** L1, the length of femur fixed by two screws closest to the fracture line; **b** L2, the length of femur fixed by the two screws farthest from the fracture line, **c** L3, the total length from the greater trochanter to the distal end of femur

Many parameters were included as potential risk factors for evaluation. Age, body weight, sex, left/right side, AO Pediatric Comprehensive Classification of Long Bone Fractures (PCCF) classification types 32-D/4.1, 32-D/4.2, 32-D/5.1, and 32-D/5.2 [16, 17], open/closed fracture, fracture location (upper, middle, or lower third of femur), open/closed reduction, operation time, perioperative bleeding, diameter and number of screws, immobilization time, and proportion of femur fixed by screws were recorded. The proportion of femur fixation was recorded as L1/L3 and L2/L3; where L1 is the length of femur fixed by the two screws closest to the fracture line, L2 is the length of femur fixed by the two screws farthest from the fracture line, and L3 is the total length from the greater trochanter to the distal end of femur (Fig. 1).

The study was approved by our children’s hospital’s ethic committee. Informed consent was signed, and all the clinical data were authorized to use for publication by the patients’ guardians.

## Statistical analysis

SPSS 17.0 software (SPSS Inc, Chicago, USA) was used to evaluate the statistical difference. Data between refracture and non-refracture groups were compared by Fisher’s exact or chi-square test and Student’s *t*-test. Logistic regression analysis was used for multivariate analysis. A *P*-value < 0.2 on univariate analysis was defined for inclusion in regression analysis. *P*-value < 0.05 was defined as statistical difference.

## Results

There were 108 male and 57 female patients, aged 8.4 ± 2.0 years (weight: 24.3 ± 10.8 kg) in this study. There were 51 upper-, 108 middle-, and 6 lower-third femur fractures. According to the PCCF classification, 138 patients had type 32-D/4.1 and 32-D/5.1 fractures and 27 patients had type 32-D/4.2 fractures. Gustilo–Anderson type I open fractures were present in 3.6% of the cases. Patient characteristics and fracture fixation details are presented in Table 1. All patients underwent open or closed reduction and EF after admission. EF was removed after bone healing, as demonstrated by disappearance of the fracture line on the radiograph and recovery of usual daily activities [14]. The average EF fixation time was 6.82 ± 2.6 months. Follow-up time was 19.7 ± 3.3 months. All surgeries were performed by attending doctors who have been trained in the pediatric orthopedic department for at least 5 years. There was no significant difference between these groups in the surgery performed by the doctors.

**Table 1:** Comparison of demographic and clinical characteristics between patients with and without refracture

Characteristics	Refracture (*n* = 24)	No refracture (*n* = 141)	*P* value
Age (years)	7.6 ± 2.2	8.5 ± 2.0	0.2512
Body weight (kg)	25.1 ± 9.6	24.2 ± 11.1	0.8376
Gender (male/female)	18/6	90/51	0.8169
Left/right side	9/15	75/66	0.3572
Open/closed fracture	3/21	8/133	0.2023
Fracture location			0.1535
Upper third	3	38	
Middle third	21	97	
Lower third	0	6	
Open/closed reduction	24/0	129/12	0.2176
Operation time (min)	25.1 ± 9.6	29.1 ± 14.7	0.7762
Perioperative bleeding (ml)	2.5 ± 1.6	3.9 ± 4.6	0.4021
Diameter of the screws			0.2598
3.5 mm	18	89	
5.0 mm	6	52	
Number of screws			0.3318
4	18	96	
5	0	12	
6	6	33	
EF fixation time (months)	6.91 ± 1.73	6.81 ± 2.81	0.9512
Immobilization time (months)	1.89 ± 0.55	2.01 ± 0.63	0.7325
Immobilization time of refracture (months)	3.13 ± 0.71	N/A	

*EF* external fixation, *N/A* not applicable

**Fig. 2:**
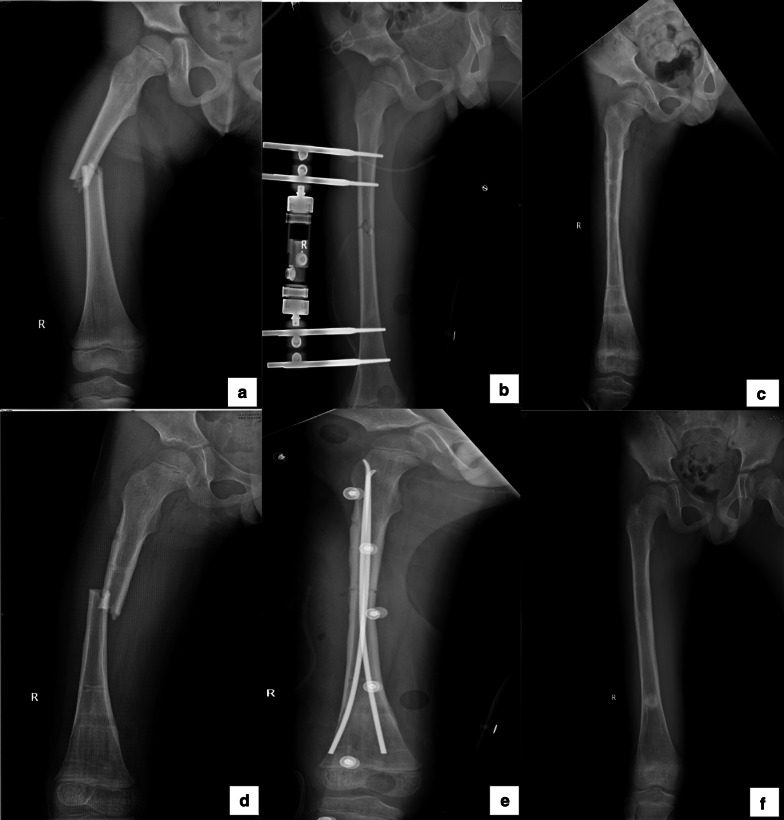
Radiograph showing the right femoral shaft fracture in a 6-year-old male patient. **a** Initial fracture; **b** Fracture treated with external fixation (EF); **c** Removal of EF after 8 months; **d** At the fourth week after the fixation removal, refracture, unfortunately, happened due to a fall; **e** Refracture treated with an elastic intramedullary nail; **f** Removal of the fixator after 11 months. No refracture occurred within 1 year

There were 24 refracture patients (14.5%; 18 male and 6 female) within 1 year after the removal of EF. Overall, 18 refractures were related to a fall injury, 3 refractures were due to the patient walking too early after EF removal, and the remaining 3 refractures were caused by the patient rolling over while sleeping. Refracture occurred time was 2.3 ± 1.2 months (range 1–5 months), calculated from the time point of EF removal. Figure 2 shows one of three typical cases in which refracture occurred within 1 month.

Univariate analysis revealed that age, body weight, sex, left/right side, open/closed fracture, fracture location, open/closed reduction, operation time, perioperative bleeding, diameter or number of screws, L1/L3 ratio, and immobilization time had no significant correlation, but PCCF fracture classification 32-D/4.2 and L2/L3 ratio (*P* < 0.001 and *P* = 0.0141, respectively) had a statistical difference on refracture (Tables 1, 2).

**Table 2:** Multivariate analysis of risk factors associated with femoral shaft refracture

Risk factors	Refracture	No refracture	*P* value
PCCF type			< 0.001^#^
32-D/4.1	5	73	
32-D/5.1	7	53	
32-D/4.2	12	15	
32-D/5.2	0	0	
L1/L3^a^	0.46 ± 0.05	0.41 ± 0.06	0.0727
L2/L3^a^	0.65 ± 0.04	0.59 ± 0.06	0.0141^#^

PCCF, AO Pediatric Comprehensive Classification of Long Bone Fractures

^#^Significant difference at *P* < 0.05

^a^L1, length of femur fixed by the two screws closest to the fracture line; L2, length of femur fixed by the two screws farthest from fracture line; L3, total femur length

## Discussion

We focused on the most severe complication, viz. refracture, analyzed the risk factors, and found that the PCCF classification type 32-D/4.2 and L2/L3 ratio were related to refracture. All patients were treated with unilateral external fixators (Orthofix^®^, Verona, Italy). EF has historically been used for temporary fixation of long bone fracture before definitive treatment by plating or EIN [18, 19]. Gradually, their use has been expanded to long-term fixation [20, 21]. At present, EIN is considered the first choice for most pediatric femoral shaft fractures, and it offers many advantages and fewer complications. Chen et al. performed a metaanalysis and reported that EIN was superior to EF for early treatment of FSF by comparing the leg length discrepancy, nail irritation, knee function, bone refracture, bone healing time, and operation time [22]. Many other studies, including some from trauma multicenters, have also reported that EIN is much more widely used than EF, although there was no significant difference between the EIN and EF methods in those studies [2, 7, 23, 24]. However, in some patients, such as those weighing over 50 kg and those with open fractures combined with other fractures and severe soft tissue damage, EF was sometimes one of the choices for fracture fixation [2, 22, 25]. In comparison with the EIN fixation method, the EF method offers much shorter immobilization time, early mobilization, and shorter hospitalization. In comparison with EIN, EF is associated with slightly higher rates of complications such as infection, refracture, and dysfunction of the knee joint [26]. Although this technique is easy to perform and most patients have good or excellent results [11], there is a consistent concern regarding complications such as refracture, infection, and knee function for patients treated with EF. However, infection and knee function may be avoided by antibiotics and function exercise; thus, the most severe complication is refracture. In our study, we found that femoral shaft refracture occurred in 24 (13.2%) of 165 children, which is similar to the findings of previous reports [1, 11]. Univariate analysis revealed that PCCF classification type 32-D/4.2 and L2/L3 ratio (*P* < 0.001 and *P* = 0.0141, respectively) were significantly associated with refracture. We speculated that the L2/L3 ratio should be within a specific range: if the ratio is too high, it may affect osteoblastic differentiation and callus formation during fracture healing, increasing the risk of delayed union or nonunion of fractures; if the ratio is too low, it could lead to overconcentration of stress at the fracture site.

Kesemenli reported a series of femoral shaft fractures in children [13], where refracture occurred in only 1 (1.8%) of 57 patients treated with closed reduction followed by EF but in 7 (20%) of 35 patients treated with open reduction. The authors, therefore, suggested that the cause of refracture was open reduction. In our study, open reduction was not associated with refracture, possibly because most of the patients underwent open reduction as most fractures in our study had shortened shafts that made closed reduction difficult.

Miner [15] revealed that open fracture was also associated with refracture, with an incidence rate as high as 20% after open fracture. Open fracture is often accompanied by severe soft tissue injury and even blood vessel damage. It has been reported that open fracture is associated with prolonged time to union compared with closed fracture [27, 28]. However, in this study, no statistical correlation was founded between union time and open fracture, the same as closed fracture and open fracture (Gustilo’s classification type 2), which may be because there were just two patients with open fracture in this study.

This study has some limitations. First, there were no patients with femoral shaft fracture of PCCF classification type 32-D/5.2, which is probably associated with refracture. Second, there were just 165 patients in our study, and the number of cases of refracture was small; therefore, more patients are needed for a more rigorous and adequately powered randomized controlled trial for verification. The evidence level of this study was low because it was a retrospective study, and a prospective or multicenter study may be needed for verification. Fourth, some other complications such as lower limb discrepancy or pin end irritation and even a longer follow-up duration were not considered in this study. Finally, a control group or another fixation material such as EIN is needed for further studies.

## Conclusions

Refracture is a major complication of pediatric femoral shaft fractures treated with EF. Because of the limited number of cases in this study, we cautiously conclude that PCCF classification type 32-D/4.2 and L2/L3 ratio were independent risk factors for refracture. We recommend that children with femoral fractures of PCCF classification type 32-D/4.2 use a protective brace for 1–2 months after EF removal and delay exercise as a precaution against secondary trauma and injury. While further studies are needed to determine the optimal L2/L3 ratio for screw placement, surgeons should be aware of the risks of placing screws either too close or too far from the fracture line.

## Data Availability

The data and material of this study are available on reasonable request to the corresponding author.
